# Therapeutic Prospects of Extracellular Vesicles in Cancer Treatment

**DOI:** 10.3389/fimmu.2018.01534

**Published:** 2018-07-03

**Authors:** Daria S. Chulpanova, Kristina V. Kitaeva, Victoria James, Albert A. Rizvanov, Valeriya V. Solovyeva

**Affiliations:** ^1^Institute of Fundamental Medicine and Biology, Kazan Federal University, Kazan, Russia; ^2^School of Veterinary Medicine and Science, University of Nottingham, Nottingham, United Kingdom

**Keywords:** extracellular vesicles, tumor microenvironment, tumor cells, immune cells, stromal cells, vaccination, cancer therapy

## Abstract

Extracellular vesicles (EVs) are released by all cells within the tumor microenvironment, such as endothelial cells, tumor-associated fibroblasts, pericytes, and immune system cells. The EVs carry the cargo of parental cells formed of proteins and nucleic acids, which can convey cell-to-cell communication influencing the maintenance and spread of the malignant neoplasm, for example, promoting angiogenesis, tumor cell invasion, and immune escape. However, EVs can also suppress tumor progression, either by the direct influence of the protein and nucleic acid cargo of the EVs or *via* antigen presentation to immune cells as tumor-derived EVs carry on their surface some of the same antigens as the donor cells. Moreover, dendritic cell-derived EVs carry major histocompatibility complex class I and class II/peptide complexes and are able to prime other immune system cell types and activate an antitumor immune response. Given the relative longevity of vesicles within the circulation and their ability to cross blood–brain barriers, modification of these unique organelles offers the potential to create new biological-tools for cancer therapy. This review examines how modification of the EV cargo has the potential to target specific tumor mechanisms responsible for tumor formation and progression to develop new therapeutic strategies and to increase the efficacy of antitumor therapies.

## Introduction

**Figure 1:**
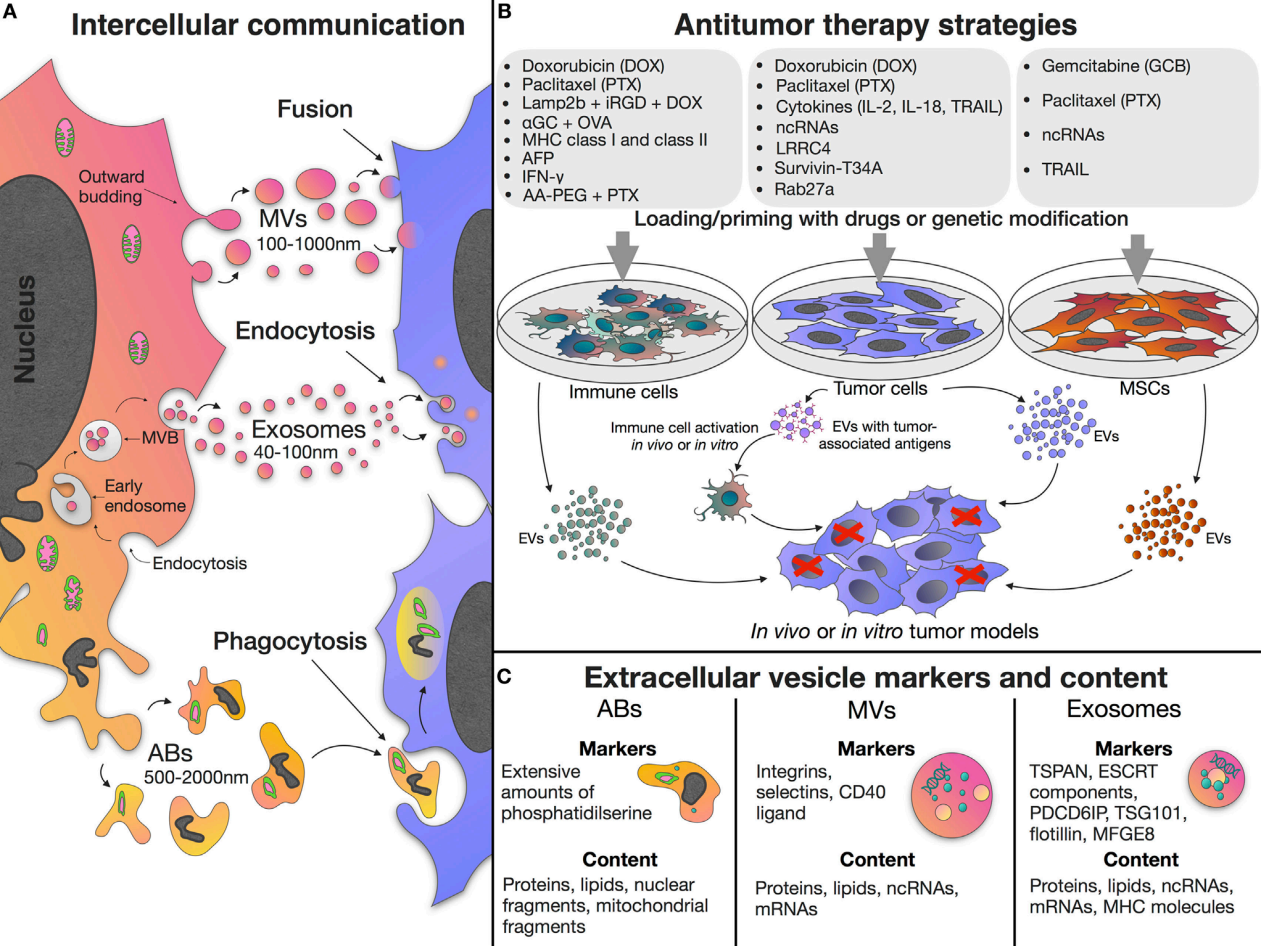
Extracellular vesicle (EV) properties and application in antitumor treatment. **(A)** EVs can be classified based upon their biogenesis and are divided into exosomes, microvesicles (MVs), and apoptotic bodies (ABs). Exosomes are formed as part of the endocytic pathway by inward budding of endosomal membranes, resulting in accumulation of early endosomes and formation of large multivesicular bodies (MVBs) which release their contents (exosomes) into the extracellular space. MVs are released by directly budding from the plasma membrane. ABs are formed as part of the fragmentation process of cells undergoing apoptosis. **(B)** EVs derived from native or primed/genetically modified cells can be used in antitumor treatment. **(C)** Different types of EVs contain various proteins, lipids, and nucleic acids and have specific membrane markers. Exosomes have tetraspanin (such as TSPAN29 or TSPAN30), endosomal sorting complex required for transport (ESCRT) components, milk fat globule-EGF factor 8 protein (MFGE8), programmed cell death 6 interacting protein (PDCD6IP), tumor susceptibility gene 101 protein (TSG101), and flotillin molecules on their surface. Exosome content include mRNAs, microRNAs, and other non-coding RNAs (ncRNAs), cytoplasmic and membrane proteins including receptors and major histocompatibility complex (MHC) molecules. MVs carry integrins, selectins, and CD40 ligand on their surface, and also contain mRNAs, microRNAs, ncRNAs, cytoplasmic and membrane proteins. ABs have extensive amounts of phosphatidylserine and contain various parts of the apoptotic cell such as proteins, lipids, nuclear fragments, and cell organelles. Cargo and biogenesis of EVs have been comprehensively discussed elsewhere (8, 9).

Extracellular vesicles (EVs) are of particular interest due to their ability to mediate intercellular communication, influencing multiple cellular processes. EVs can be categorized based upon their biogenesis and divided into exosomes, microvesicles (MVs), and apoptotic bodies (ABs) (1, 2). Exosomes are small vesicles 40–100 nm in diameter, formed as part of the endocytic pathway. Exosomes carry the donor cell cargo, represented by various proteins and nucleic acids [DNA, mRNA, miRNA, and other non-coding RNAs (ncRNAs)] (Figure 1C) (3, 4). Exosomes are stable in biological fluids and small enough to pass through the blood–brain barrier (5). MVs have a diameter of 100–1,000 nm and are released by directly budding from the plasma membrane (6). MVs also carry cargos of proteins and nucleic acids, although their functional roles in cell-to-cell communication remains less well studied than the exosome population (7). In contrast to exosomes and MVs, which are formed continuously by cells, ABs are formed as part of the fragmentation process of cells undergoing apoptosis, the process of programmed cell death (1) (Figure 1A).

The tumor microenvironment is often a very complex and dynamic niche containing not only neoplastic cells but also a multitude of non-malignant stromal cells such as endothelial cells, tumor-associated fibroblasts, pericytes, and immune cells (10). In addition to stromal cells, the extracellular matrix and surrounding tumor adipose tissue also make an important contribution to tumor progression as they contain adipocytes and progenitor cells [preadipocytes and mesenchymal stem cells (MSCs)] (10, 11), as well as a variety of soluble cytokines, growth factors, and metabolites produced the stromal cells within the tumor microenvironment (10, 12). As EVs are believed to mediate cell-to-cell communication in the tumor microenvironment and induce phenotypic modification in recipient cells, there is a growing interest in the potential role of EVs as key mediators of tumor progression and the spread of malignant neoplasm (13–16). Since EV functions are related to the donor cell type and the imparted cargo of proteins and nucleic acids, EVs of different origins exhibit different features. However, as these have been comprehensively discussed elsewhere (17), this review focuses on the use and efficacy of EVs as antitumor therapies. For instance, as a result of the unique properties of MSCs, the EVs produced by stem cells retain the ability to migrate toward tumor niches (18), they also posses the same low immunogenicity of the donor MSCs (19). Therefore, the use of MSC-derived EVs as non-cell structures, in place of MSCs themselves, allows the avoidance of the risk of unlimited cell growth, undesirable transformation, and potential tumor formation (20). The ability to act as multi-signal messengers makes EVs a prospective new class of therapeutic agents to modulate the processes occurring in the tumor microenvironment (21) (Figure 1B).

## Tumor Cell-Derived EVs

Intercellular EV-mediated signaling by tumor cells has been linked with maintain angiogenesis, invasion, immune escape (22) and to develop an aggressive phenotype and chemo- and radiotherapy resistance (16, 23–25). The extent of the contribution of EVs in tumor maintenance has been demonstrated through the study of EV inhibition, following which malignancy is suppressed and cancer cells show enhanced sensitivity to proton-pump inhibitor (omeprazole) and cisplatin (26, 27). As EV traffic is regulated by an acidic microenvironment, a common feature of all solid tumors, altering intracellular pH is an effective means of modulating exosome release. Changes in intracellular pH alters the lipid composition of the cells membrane and subsequently modulates both exosome release and fusion/uptake (28). In addition, the lower extracellular pH can promote tumor resistance to cytotoxic drugs through neutralization of those antitumor drugs that are weak bases or isolating drugs in acidic vesicles and/or eliminating them through an exocytotic pathway (29).

Extracellular vesicles may also promote tumor progression through the transfer of their specific cargos, for example, during the formation of a pre-metastatic niche (PMN), where the transfer of EV-cargos to stromal cells, induce molecular and cellular changes that promote PMN development (30, 31). For example, the tumor exosomal transport of miR-494 and miR-542p to stromal cells and lung fibroblasts leads to cadherin-17 downregulation and matrix metalloproteinase upregulation (30), while proangiogenic RNAs contained within MVs trigger angiogenesis to promote PMN formation (32).

The ability of tumor cell-derived EVs to fuse with recipient cells through endocytosis and release their cargo into the recipient cell cytoplasm makes EVs a promising biological vector for targeted delivery of various antitumor agents (33). This is exemplified by the use of EVs derived from LNCaP and PC-3 prostate cancer cell lines modified to transport paclitaxel (PTX) into recipient cells through the endocytic pathway, significantly increasing PTX cytotoxicity *in vitro* (33). Furthermore, U-87 MG (brain neuronal glioblastoma–astrocytoma) derived EVs primed with doxorubicin (DOX) or PTX significantly decreased the viability of recipient U-87 MG cells by 70 and 50%, respectively, at the highest tested concentration of exosomes (200 µg/mL) *in vitro* (34).

Tumor-derived EVs can be used for therapeutic drug delivery to reduce systemic toxicity by targeting the tumor microenvironment. It was shown that *in vitro* and *in vivo*, doxorubicin-loaded exosomes (exoDOX) derived from MDA-MB-231 (breast adenocarcinoma) and HCT-116 (colorectal carcinoma) cell lines did not reduce DOX efficacy. Simultaneously, exoDOX treated nude mice did not show the cardiotoxicity observed in their free-DOX-treated counterparts. Mass spectrometry confirmed that DOX accumulation in the heart was reduced by approximately 40% when DOX was delivered *via* exosomes (exoDOX) (35). The reduced cardiotoxicity achieved when delivering DOX *via* modified exosomes would allow for a higher concentration of exoDOX to be used, thus offering the potential to increase DOX efficacy. Similar findings have also been reported for *in vivo* models of breast (MDA-MB-231) and ovarian (STOSE) cancer (36).

Tumor cell-derived EVs carry on their surface the same antigens as the cell that produced them (the donor cell), such as HER2/*neu*, melan-A, Silv, carcinoembryonic antigen (CEA), mesothelin, and others (37). Thus, they can act to prime immune cells by antigen presentation. The delivery of dendritic cells (DCs) *in vitro* primed with exosomes isolated from the mesothelioma cell line AB1 within a BALB/c mouse mesothelioma model, resulted in increased mean and overall survival times *in vivo* (38). Similarly, DCs primed with exosomes isolated from rat glioblastoma cells, induced a strong antitumor response and significantly increased median survival times in glioblastoma-bearing rats when used in combination with α-galactosylceramide (39).

The efficacy of priming immune cells can be improved by combining their use with immune cell stimulating drugs. For instance, exosomes derived from the pancreatic cancer cell line UNKC6141 were co-delivered with DCs (DCs/Exo) to UNKC16141 xenograft mice. Tumor onset was delayed in these animals and subsequently a significant increase in survival was observed. When the same assay was repeated, but with the inclusion of all-transretinoic acid (ATRA) alongside the delivery of DCs/Exo, increased lymphocyte proliferation within lymph nodes was reported which coincided with increased cytotoxic T-cell activity in comparison with untreated or DCs/Exo only treated animals. However, the inclusion of ATRA had no further effect on prolonging survival and only modest changes in metastasis to distant organs were observed. The combination of DCs/Exo with sunitinib in these animal models also led to an increase in cytotoxic activity which in these assays did lead to significantly prolonged survival times in DCs/Exo/sunitinib compared to animals treated only with free sunitinib therapy. Similar increases in survival time and a reduction in metastatic spread was also observed when DCs/Exo use was combined with gemcitabine treatment (40).

To increase the therapeutic potential and immunogenicity of EV-based tumor vaccines, tumor cells producing the EVs can be modified to express specific cytokine/chemokine genes that have an immunomodulating effect. Dai et al. reported that exosomes derived from LS-174T cells genetically modified to express IL-18 CEA (Exo/IL-18), had a more pronounced effect on specific antitumor immunity when compared with exosomes from native LS-174T cells. Exo/IL-18 promoted proliferation of peripheral blood mononuclear cells and induced cytokine secretion by T-lymphocytes and DC *in vitro*, as well as inducing the phenotypic and functional maturation of DCs (41). Similar results were obtained by Yang et al. using *in vivo* experiments, whereby exosomes were derived from IL-2-modified ovalbumin (OVA)-expressing EL-4 lymphoma cells (Exo/IL-2). Vaccination of C57BL/C mice with Exo/IL-2 more effectively inhibited tumor growth (42).

The modification of tumor cells through the aberrant expression of tumor suppressor genes, apoptosis inductors, and ncRNAs has also been shown to impart a potential therapeutic benefit to the resulting EVs. YUSAC 2 melanoma cells were engineered to overexpress a dominant-negative mutant form of Survivin (Survivin-T34A). Exosomes derived from Survivin-T34A-modified YUSAC 2 cells, in combination with gemcitabine, significantly increased apoptosis in pancreatic adenocarcinoma MIA PaCa-2 cells in comparison with gemcitabine alone (43). Rivoltini et al. showed that exosomes derived from K562 leukemia cells modified with TNF-related apoptosis-inducing ligand (TRAIL) [TRAIL(+) exosomes], induced apoptosis in TRAIL-death receptor (DR)5(+) SUDHL4 lymphoma and INT12 melanoma cells *in vitro*. In *in vivo* experiments of TRAIL(+) exosomes demonstrated homing of the exosomes to the tumor sites and significant suppression of tumor growth by 58% in SUDHL4-B-cell lymphoma bearing mice (44). Li et al. investigated exosomes derived from glioblastoma multiforme (GBM) cells with overexpression of the tumor suppressor gene LRRC4 (Exo/LRRC4). Exo/LRRC4 induced significant chemotaxis and expansion of CD4^+^CCR4^+^ T cells, inhibited the proportion of Ti-Treg cells, and promoted Ti-Teff cell expansion through cytokines release *in vitro* (45).

The Rab GTPases control many stages of membrane trafficking, including the formation and release of vesicles. Ostrowski et al. identified Rab GTPases Rab2b, Rab9a, Rab5a, Rab27a, and Rab27b that promote exosome secretion in HeLa cells (46), indicating the possibility of manipulating the secretion of Rab proteins to control exosome production. Exosomes, derived from Rab27a-overexpressing A549 cells (exo/Rab27a), exhibited the ability to regulate major histocompatibility complex (MHC) class II molecules and co-stimulatory molecules CD80 and CD86 on DCs. Furthermore, DCs primed with exosomes derived from Rab27-overexpressing A549 cells significantly increased CD4^+^ T cell proliferation *in vitro*. *In vivo* immunization with exo/Rab27a inhibited tumor growth in a tumor mouse model (47).

At present ncRNAs are actively being studied as potential antitumor agents. However, when developing miRNA-based therapies there are problems with specific targeting of tumor cells and target cells within the tumor microenvironment. Tumor-derived EVs can be used for delivering a variety of potentially therapeutic ncRNAs, for instance miR-134 (48), miR-29a, and miR-29c microRNAs (49), as well as short interfering RNAs (siRNAs) (50) (Table 1).

**Table 1:** The use of extracellular vesicles (EVs) with or without modified cargo for antitumor therapy.

Vesicle source	Vesicle type	Purification strategy	Cargo	Mechanism of action	Model	Reference
**Cancer cells**						

Glioblastoma–astrocytoma U-87 MG cells	Exosomes	Exosome isolation reagent (Invitrogen)	DOX or PTX	Cell viability decrease	*In vitro* U-87 MG cell culture	(34)

LNCaP and PC-3 prostate cancer cells	Exosomes and microvesicles	Differential centrifugation	PTX	PTX cytotoxic effect increase	*In vitro* PC-3 and LNCaP cell culture	(33)

MDA-MB-231 and HCT-116 cell lines	Exosomes	ExoQuick-TC™ solution (System BioSciences)	DOX	Cardio toxicity decrease, DOX efficacy increase	MDA-MB-231 cell mice model *in vivo*	(35)
	
MDA-MB-231 and STOSE cell lines	Exosomes	AB cell culture-nanovesicles solution (AB ANALITICA)	DOX	Breast MDA-MB-231 and ovarian STOSE mouse tumors *in vivo*	(36)

Oral cancer cells	Exosomes	Ultrafiltration and affinity chromatography	Tumor-associated antigens	NK cell proliferation and NK cell cytotoxicity increase	*In vitro* NK cell culture	(51)

Mouse malignant mesothelioma (MM) AB1 cells	Exosomes	Stepwise ultracentrifugation	Tumor-associated antigens	Exosome-loaded dendritic cell (DC) increased median and overall survival	AB1 tumor BALB/c mice model *in vivo*	(38)

Rat glioblastoma	Exosomes	ExoRNeasy Serum/Plasma Maxi Kit (Qiagen)	Tumor-associated antigens + α-galactosylceramide	Exosomes pulsed DCs increased median survival time	Glioblastoma-bearing rat model *in vivo*	(39)

UNKC6141 (pancreatic cancer) cells	Exosomes	Sucrose gradients ultracentrifugation	Tumor-associated antigens	Exosome-loaded DCs delayed tumor onset and increased survival time	UNKC6141-bearing mice	(40)
				DCs/Exo + all-transretinoic acid increased proliferation of lymph node cells and cytotoxic T cell activity		
				DCs/Exo and sunitinib prolonged survival time		
				DCs/Exo + gemcitabine prolonged survival time		

Carcinoembryonic antigen (CEA)-expressing LS-174T tumor cells	Exosomes	Sucrose gradients ultracentrifugation	IL-18	Maturation of DCs and induction of CEA-specific CD8^+^ CTL	DCs and CTL cells *in vitro*	(41)

OVA-expressing EL-4 lymphoma cells	Exosomes	Sucrose gradients ultracentrifugation	IL-2	Immune response induction and tumor growth inhibition	C57BL/C mice model *in vivo*	(42)

YUSAC 2 melanoma cells	Exosomes	Sucrose gradients ultracentrifugation	Survivin-T34A (Survivin blocking protein)	Caspase activation and apoptosis induction	Pancreatic cancer cells *in vitro*	(43)

K562 leukemia cells	Exosomes	Differential centrifugation	TNF-related apoptosis-inducing ligand (TRAIL)	TRAIL-related apoptosis induction	SUDHL4 lymphoma and INT12 melanoma cells *in vitro*	(44)

				Tumor growth inhibition	SUDHL4-bearing mice	

A549 cells	Exosomes	Differential centrifugation	Rab27a	Maturation of major histocompatibility complex (MHC) class II molecules, CD80 and CD86. Inhibition of tumor growth	DCs *in vitro*, BALB/c mice model *in vivo*	(47)

Glioblastoma multiforme (GBM) cells	Exosomes	Differential centrifugation	LRRC4	Chemotaxis and expansion of CD4^+^ CCR4^+^ T cells	GBM cells *in vitro*	(45)

Hs578T and Hs578Ts(i)8 cells	Exosomes	Filtration and ultracentrifugation	miR-134	Cellular migration and invasion reduction, drugs sensitivity enhancement	Hs578Ts(i)8 cells *in vitro*	(48)

SGC7901 cells	Microvesicles	Differential centrifugation	miR-29a and miR-29c	Angiogenesis and tumor growth suppression	Implanted with SGC7901 cells BALB/c mice *in vivo*	(49)

HeLa and HT1080 cells	Exosomes	Differential centrifugations and micro-filtration	Short interfering RNAs (siRNAs) against RAD51 and RAD52	Accumulation of the cells in S and G2/M phases of cell cycle and recipient cell death induction	HeLa cells *in vitro*	(50)

**Immune cells**						

DCs	Exosomes	Sucrose gradients ultracentrifugation	Lamp2b + iRGD + DOX	Tumor growth inhibition	MDA-MB-231 injected BALB/c nude mice model *in vivo*	(52)

DCs	Exosomes	Differential centrifugation	αGC + OVA	NK and γδ T-cell immune responses induction	Invariant NKT cells *in vitro*	(53)

				Tumor growth decrease	B16/OVA melanoma tumor model *in vivo*	

DCs	Exosomes	Ultrafiltration/diafiltration and sucrose gradients ultracentrifugation	MHC class I and class II	NK cell proliferation and activation, IFNγ secretion enhancement	NK cells *in vitro*	(54)

			MHC class I and class II	NK cell proliferation and activation by trans-presentation of IL-15 by IL-15Rα, number of metastases reduction	Mouse model *in vivo*	

DCs	Exosomes	Differential centrifugation	AFP	Survival rate prolongation	Tumor-bearing C57BL6 mice model *in vivo*	(55)

DCs	Exosomes	Ultrafiltration/diafiltration and sucrose gradients ultracentrifugation	IFN-γ	NKp30-dependent NK cell function enhancement	Advanced non-small cell lung cancer patients	(56)

RAW 264.7 macrophages	Exosomes	ExoQuick-TC™ solution (System BioSciences)	PTX	Drug cytotoxicity increase, inhibition of metastases growth	Resistant multidrug resistance cell culture *in vitro*, Lewis lung carcinoma mouse model *in vivo*	(57)

			AA-PEG + PTX	Suppression of metastases growth and survival time increase	*In vivo* C57BL/6 mice lung cancer model	(58)

Monocytes or macrophages	Exosome-mimetic nanovesicles	Iodixanol gradients ultracentrifugation	DOX	Apoptosis increase and number of proliferating cells reduction	*In vivo* model of mouse CT26 colorectal cancer	(59)

**Mesenchymal stem cells (MSCs)**						

MSCs	Exosomes	Differential centrifugation	Anti-miR-9	Temozolomide sensitivity increase	Temozolomide-resistant GBM cell culture *in vitro*	(60)

MSCs	Exosomes	ExoQuick-TC™ solution (System BioSciences)	miR-146b	Tumor growth reduction	*In vivo* rat model of primary brain tumor	(61)

MSCs	Exosomes	Sucrose gradients ultracentrifugation	miR-124a	Viability and clonogenicity reduction	Glioma stem cell lines *in vitro*	(62)

				Prolonged survival rate	*In vivo* model of mouse GSC267 glioma	

Bone marrow MSCs (BM-MSCs)	Exosomes	ExoQuick-TC™ solution (System BioSciences)	miR-340	Tumor angiogenesis inhibition *via* the HGF/c-MET signaling pathway	Endothelial cell culture *in vitro*	(63)

MSCs	Exosomes	Differential centrifugation	Polo-like kinase 1 (PLK-1) siRNA	Cancer cell proliferation reduction by PLK-1 gene silencing	Bladder cancer cells *in vitro*	(64)

MSCs	Exosomes	ExoQuick-TC™ solution (System BioSciences)	miR-122	Antitumor efficacy of sorafenib increase	Hepatocellular carcinoma model *in vivo*	(65)

BM-MSCs	Microvesicles	Differential centrifugation	PTX	Tumor growth inhibition	Human pancreatic adenocarcinoma CFPAC-1 cells *in vitro*	(66)

MSCs	Microvesicles	Differential centrifugation	PTX or GCB	Tumor proliferation inhibition	Pancreatic cancer cells *in vitro*	(67)

MSCs	Exosomes	Sequential ultracentrifugation combined with 0.22 µm ultrafiltration	TRAIL	Apoptosis induction	M231 breast cancer cells and other cancer cell lines *in vitro*	(68)

## Immune Cell-Derived EVs

Exosomes from immature dendritic cells (imDCs) can be used to deliver chemotherapeutic agents such as DOX. For instance, imDCs were modified to express lysosome-associated membrane protein 2 (Lamp2b) fused to the αv-integrin-specific iRGD peptide. It was shown that modified imDC-derived exosomes (Exo/iRGD) loaded with DOX, effectively targeted and delivered DOX to αv-integrin^+^ MDA-MB-231 breast cancer cells *in vitro*. Exo/iRGD intravenous injection in BALB/c mice led to inhibition of breast tumor cell growth without any apparent toxic effects (52).

A new approach for cancer immunotherapy is the combination of exosomes and the invariant NKT immune cell ligand α-galactosylceramide (αGC) (53). Loaded with αGC and OVA-model antigen exosomes induced potent NK and γδ T-cell innate immune responses *in vitro* and *in vivo*. In an OVA-expressing mouse model of melanoma treatment of tumor-bearing mice with αGC/OVA-loaded exosomes decreased tumor growth, increased antigen-specific CD8^+^ T-cell tumor infiltration, and increased median survival, relative to control mice immunized with soluble αGC + OVA alone (53). Similarly, exosomes derived from α-fetoprotein (AFP)-expressing DCs (DEXAFP) intravenously injected into hepatocarcinoma-bearing C57BL6 mice prolonged survival to 57 days in 100% of DEXAFP-treated mice (55).

Without modification, DC-derived exosomes alone carry MHC class I and class II/peptide complexes capable of leading to the priming of CD8^+^ and CD4^+^ T cells, respectively, and subsequent T cell-dependent tumor rejection (13, 54). DC-derived exosomes have also been reported to trigger NK cell proliferation and activation *in vitro* and in patients, by trans-presentation of IL-15 by IL-15Rα. This mechanism of action was shown to significantly reduce the number of lung metastases *in vivo*. Combination of DC-derived exosomes with IL-15Rα and rhIL-15 molecules led to NK cell proliferation and activation and significantly enhanced IFNγ secretion by NK cells *in vitro* (54).

Phase I clinical trials have demonstrated the safety of using DC-derived exosomes in patients with metastatic melanoma (69) and lung cancer (70). Phase II trials in non-small cell carcinoma patients using modified IFN-γ expressing DCs to produce exosomes have reported an increase in NKp30-dependent NK cell functions, and 32% of participants experienced stabilization for more than 4 months (56).

In addition to DCs, macrophages have also been studied as a source of EVs of potential therapeutic benefit. Derived from RAW 264.7 macrophages, vesicles loaded with PTX (exoPTX) were reported to significantly increase drug cytotoxicity (more than 50 times) in multidrug resistance (MDR) MDCKMDR1, MDCKwt, and 3LL-M27 cells *in vitro*. Furthermore, when delivered into the airway of mice modeling Lewis lung carcinoma pulmonary metastases, exoPTX were found to have a potent anticancer effect (57). For PTX targeted delivery macrophages can be modified with aminoethylanisamide-polyethylene glycol (AA-PEG) a vector moiety to target the σ-receptor which is overexpressed by lung cancer cells (58). Jang et al. developed a bioinspired exosome-mimetic nanovesicles that can be modified to deliver DOX, gemcitabine, or carboplatin to the tumor tissue after systemic administration. Chemotherapeutic-loaded nanovesicles, derived from monocytes or macrophages, induced TNF-α-stimulated endothelial cell (HUVECs) death in a dose-dependent manner *in vitro*. DOX-loaded nanovesicles increased apoptosis and reduced the number of proliferating cells in CT26 colorectal cancer murine models (59) (Table 1).

## MSC-Derived EVs

Extracellular vesicles released from MSCs have been reported to exhibit variable effects on tumor growth, indicating the influence of EVs is dependent on cargo and the donor cell type (71, 72). Delivered by MSC-derived exosomes molecules of different types of RNA can induce adipogenesis, angiogenesis, apoptosis, and proteolysis in recipient cells (15). Exosomes from gastric cancer-derived MSCs were found to deliver miR-221 to HGC-27 gastric cancer cells, promoting their proliferation and migration *in vitro* (73). Other biomolecules carried by exosomes such as oncogenic proteins, cytokines, adhesion molecules, and anti-apoptotic proteins can also promote tumor progression (74–76), as well as increase tumor resistance to chemotherapy drugs (77).

Exosomes from bone marrow MSCs (BM-MSCs) can transfer miRNAs from the BM, particularly miR-23b, which promote dormancy in bone marrow-metastatic human breast cancer through the suppression of a target gene, MARCKS *in vivo* (78). In support of this, Lee et al. showed that MSC-derived exosomes can suppress human breast cancer angiogenesis by downregulating the expression of VEGF in tumor cells *in vitro* and *in vivo* (79).

In addition to the endogenous effects of MSC-EVs, MSC-derived MVs can be used as delivery vehicles for a variety of potential therapeutic agents, in particular ncRNAs. For example, injection of exosomes derived from miR-146-expressing MSCs into xenograft gliomas in primary brain tumor rat models cause a significant reduction in tumor growth (61). Treatment with MSC-derived exosomes containing miR-124a reduce the viability and clonogenicity of glioma stem cell lines *in vitro* and increase the survival rate in glioma mouse models up to 50% by silencing FOXA2 (62), while the loading of MSC exosomes with miR-143 acts to significantly reduce the migration of 143B osteosarcoma cells (80). Transfection of bone marrow stromal cells with miR-340 generates exosomes capable of inhibiting tumor angiogenesis *via* the HGF/c-MET signaling pathway in endothelial cells (63). MSC-derived EVs can also be used to alter the chemosensitivity of tumor cells. Delivery of anti-miR-9 to temozolomide-resistant GBM cells increases cell sensitivity to this drug (60). The sensitivity of hepatocellular carcinoma cells to chemotherapeutic agents (5-fluorouracil and sorafenib) can similarly be altered through the use of miR-122 loaded MSC exosomes *in vivo* (65). MSC-derived MVs can also be loaded with various siRNAs that target key genes driving tumorigenesis, for example, MSC exosomes carrying siRNAs against polo-like kinase 1 significantly reduce bladder cancer cell proliferation *in vitro* (64).

In addition to biomolecules, MSC-derived vesicles can be loaded with chemotherapeutic drugs. BM-MSC-derived MVs primed with high-dose PTX inhibited cell growth by 50% in human CFPAC-1 pancreatic adenocarcinoma cells *in vitro* (66). This finding was supported by the recent studies of Cocce et al., which showed antitumor activity of MSCs MVs loaded with PTX or gemcitabine (GCB) on pancreatic cancer cells *in vitro* (67).

Recent studies have also highlighted the potential to deliver TRAIL by MSC-EVs (MSCT). MSCT-EVs induced apoptosis in 11 cancer cell lines in a dose-dependent manner but showed no cytotoxicity in human bronchial epithelial cells *in vitro*. Interestingly TRAIL-primed EVs that contain 3.88 ng TRAIL/mL induced significantly more apoptosis in M231 breast cancer cells compared with 100 ng/mL of recombinant TRAIL. TRAIL delivery by MSC-EVs induced significant apoptosis in TRAIL resistant A549 lung adenocarcinoma cells in a dose-dependent manner *in vitro* (68) (Table 1).

## Conclusion

Extracellular vesicles, which include groups of differing origins such as exosomes and MVs, are released by all cells within the tumor microenvironment during normal cellular activity. EVs carry variable cargos that reflect the composition of the donor cells, these cargos can be transferred to neighboring cells and thus affect the processes occurring in those recipient cells and subsequently the tumor microenvironment as a whole. In addition to their endogenous ability to influence tumor progression, the ability to modify the EV content makes them a promising tool for cancer therapy. Surface antigens of tumor cell-derived vesicles can be used for immune cell priming. They can also be modified with various agents to directly affect tumor cells or modulate antitumor immunity. Genetic modifications can also be performed on MSC-derived vesicles, the main advantage of which is targeted cargo delivery to the tumor microenvironment. From priming the immune response to delivering ncRNAs and antitumor drugs, EVs provide a unique biological means of targeting tumors and their microenvironments, minimizing cytotoxic effects, and increasing the efficacy of treatments at lower drug doses (Table 1). However, despite these many advantages, EVs can have variable effects on tumor progression and the tumor microenvironment dependent upon their protein and nucleic acid cargos. One of the limitations of EV usage is the heterogeneity of the isolated population, since the size of exosomes and MVs overlap, and as yet it is not clear which population carries the greatest potential to elicit functional changes. Furthermore, the inconsistency of the EV cargo adds an additional caveat to their study and therapeutic use (81). In the case of drug loading, disadvantages include a low transfection efficiency, and, in the case of cell manipulation, there is a high dependence on cell division (82). Therefore, progressing their use as therapeutic tools requires full characterization of such disadvantages and limitations before the promise of MVs in clinical practice is achieved.

## Author Contributions

DC wrote the manuscript and made the table. KK created the figure. VJ edited the manuscript. DC, VS, and AR conceived the idea and edited the manuscript and table.

## Conflict of Interest Statement

The authors declare that the research was conducted in the absence of any commercial or financial relationships that could be construed as a potential conflict of interest.
